# AXL Inhibition Enhances MEK Inhibitor Sensitivity in Malignant Peripheral Nerve Sheath Tumors

**DOI:** 10.26502/jcsct.5079091

**Published:** 2020-10-27

**Authors:** Sharon M. Landers, Angela D. Bhalla, XiaoYan Ma, Kristelle Lusby, Davis Ingram, Ghadah Al Sannaa, Wei-Lien Wang, Alexander J. Lazar, Keila E. Torres

**Affiliations:** 1 Department of Surgical Oncology, The University of Texas MD Anderson Cancer Center, Houston, TX, USA; 2 Department of Surgery, Division of Plastic Surgery, Indianapolis University School of Medicine, Indianapolis, IN, USA; 3 Department of Pathology, The University of Texas MD Anderson Cancer Center, Houston, TX, USA; 4 Department of Pathology and Genomic Medicine, Houston Methodist Hospital, Houston TX, USA

**Keywords:** Malignant peripheral nerve sheath tumor, AXL, MEK inhibition

## Abstract

Dysregulation of the receptor tyrosine kinase AXL is known to promote cancer cell growth and survival in many sarcomas, including the rare subtype, malignant peripheral nerve sheath tumors (MPNST). MPNSTs are largely chemoresistant and carry a poor prognosis. AXL is an attractive potential therapeutic target, as it is aberrantly expressed, and its activation may be an early event in MPNST. However, the effect of AXL inhibition on MPNST development and progression is not known. Here, we investigated the role of AXL in MPNST development and the effects of AXL and MEK1/2 co-inhibition on MPNSTs. We used western blotting to examine AXL expression and activation in MPNST cell lines. We analyzed the effects of exogenous growth arrest-specific 6 (GAS6) expression on downstream signaling and the proliferation, migration, and invasion of MPNST cells. The effect of AXL knockdown with or without mitogen-activated protein kinase (MAPK) inhibition on downstream signal transduction and tumorigenesis was also examined *in vivo* and *in vitro*. We found that AXL knockdown increased MAPK pathway signaling. This compensation, in turn, abrogated the antitumorigenic effects linked to AXL knockdown *in vivo*. AXL knockdown, combined with pharmacological MEK inhibition, reduced the proliferation and increased the apoptosis of MPNST cells both *in vitro* and *in vivo*. The pharmacological co-inhibition of AXL and MEK1/2 reduced MPNST volumes. Together these findings suggest that AXL inhibition enhances the sensitivity of MPNST to other small molecule inhibitors. We conclude that combination therapy with AXL inhibitor may be a therapeutic option for MPNST.

## 1. Introduction

Malignant peripheral nerve sheath tumors (MPNSTs) are deadly soft tissue sarcomas. These tumors originate from peripheral nerves and the cell of origin is thought to be Schwann cells [1, 2]. Approximately, 50% of MPNSTs occur in the context of neurofibromatosis type 1 (NF1), 40% are sporadic and 10% develop following radiation treatment [3–5]. MPNSTs often recur, and the survival rate can be as low as ~7% [6]. Between 40% and 65% of MPNSTs recur after treatment and the 5-year survival rate of patients with these tumors ranges from 16% to 52% [7–12]. Therefore, identifying specific, targetable molecular dysregulations that contribute to malignant transformation is crucial for the development of effective therapeutic strategies.

The overexpression of receptor tyrosine kinases (RTKs) has been shown to increase the growth, survival, and metastasis of cancer cells, including MPNST cells [13]. We previously found that the receptor AXL is expressed in 91% of MPNSTs and nearly 95% of neurofibromas (the precursor lesions for MPNSTs), which suggests that AXL dysregulation is an early event in MPNST development [14, 15]. The abnormal expression and activation of AXL provides cancer cells a survival advantage by activating the phosphatidylinositol 3-kinase (PI3K)/mammalian target of rapamycin (mTOR) and MAPK pro-survival pathways as well as pathways related to cell growth, migration, and invasion [16]. Also, high AXL expression has been linked to chemoresistance in other cancers such as breast cancer, further supporting the notion that AXL expression promotes the survival of cancer cells [17]. Based on these previous studies, we hypothesized that AXL inhibition represents a new therapeutic strategy against MPSNTs.

In the present study, we examined AXL activation in an established MPNST and neurofibroma tissue microarray (TMA), patient-derived MPNST cell lines, and human MPNST patient-derived derived xenograft (PDX) models. We then investigated AXL inhibition in combination with inhibition of mitogen-activated protein kinase kinase (MEK). We observed that the long-term knockdown of AXL with short hairpin RNAs (shRNAs) in MPNSTs resulted in the compensatory upregulation of extracellular signal-regulated kinase (ERK) signaling, which in turn promoted the growth of MPNST xenografts. MEK inhibition alone reduced but was not sufficient to completely inhibit MPNST cell growth *in vivo* and *in vitro*. Combining the MEK1/2 inhibitor PD0325901 with AXL knockdown decreased the proliferation of MPNST cells *in vitro* and reduced MPNST growth *in vivo*. Our studies support that combination therapy with the AXL inhibitor BMS777607 and the MEK1/2 inhibitor PD0325901 further reduced MPNST growth *in vivo*. Together, these results suggest that the co-inhibition of AXL and MEK1/2 is a potential treatment strategy for MPNST patients.

## 2. Materials and Methods

### 2.1 Cell lines

We obtained four patient-derived MPNST cell lines: MPNST724 and ST88–14, were generously provided by Dr. Jonathan Fletcher (Brigham and Women’s Hospital, Boston, MA); STS26T, kindly provided by Dr. Steven Porcelli (Albert Einstein College of Medicine, New York, NY); and S462, generously provided by Lan Keuwe (University Medical Center Hamburg-Eppendorf, Hamburg, Germany). MPNST cells were propagated in Dulbecco’s modified Eagle’s medium (DMEM) supplemented with 10% fetal bovine serum (FBS) and 1% penicillin/streptomycin. Primary adult human normal Schwann cells (ScienCell Research Laboratories, Carlsbad, CA) were grown in Schwann cell medium (ScienCell Research Laboratories, Carlsbad, CA) and supplemented with 5% FBS and 1% penicillin/streptomycin. All cell lines were validated with DNA short tandem repeat analysis, as described previously [14].

### 2.2 Assessment of cell proliferation

Cell proliferation was evaluated using a CellTiter 96 AQueous One Solution Cell Proliferation Assay (containing 3-(4,5-dimethylthiazol-2-yl)-5-(3-carboxymethoxyphenyl)-2-(4-sulfophenyl)-2H-tetrazolium, inner salt; MTS) (Promega, Madison, WI) according to the manufacturer’s instructions. Briefly, after the cells were seeded, the medium was replaced with DMEM containing either dimethyl sulfoxide or dimethyl sulfoxide plus 800 ng/mL GAS6 [18–20]. After 96 hours (h), 3-(4,5-dimethylthiazol-2-yl)-5-(3-carboxymethoxyphenyl)-2-(4-sulfophenyl)-2H-tetrazolium (MTS) was added, the cells were incubated for two h at 37°C, and absorption at 490 nm was read using a DTX 880 microplate reader (Beckman Coulter, Brea, CA). For each experiment, proliferation was assayed in triplicate, and the percent cell proliferation was normalized to the control.

### 2.3 Modified Boyden chamber assay

Cell migration and invasion were examined with the use of modified Boyden chambers, as described previously [21]. Briefly, 2.0 × 10^5^ cells (for the migration assay) or 4.0 × 10^5^ cells (for the invasion assay) were seeded in the upper compartment of the chamber with or without GAS6 (800 ng/mL). After incubating for 16, the cells that had either migrated or invaded through the filter were fixed in 5% glutaraldehyde and stained with 0.1% crystal violet. Quantification of cells that invaded or migrated was calculated with the use of ImageJ software [22, 23].

### 2.4 Stable knockdown of AXL

AXL was stably knocked down in S462 and MPNST724 cells by transducing the cells with pGIPZ lentiviral particles containing anti-AXL shRNA either V3LHS_329651 [AXLsh1] or V3LHS_329652 [AXLsh2]; GE Healthcare, Marlborough, MA) according to the manufacturer’s instructions. Cells transduced with a non-targeting (NT) sequence (RHS4348) were used as control cells. Stably transduced cells were selected with puromycin (2 μg/mL), and knockdown of AXL was confirmed by western blotting. MPNST724-NT, MPNST724-AXLsh1, and MPNST724-AXLsh2 cells and S462-NT, S462-AXLsh1, and S462-AXLsh2 cells were maintained in normal growth medium supplemented with 2 μg/mL puromycin.

### 2.5 Western blot analysis

Whole-cell extracts were obtained by lysing subconfluent proliferating cells or tissue samples from xenografts in radioimmunoprecipitation assay (RIPA) buffer. Standard western blot protocols were used [24]. After transferring to polyvinylidene difluoride membranes, the membranes were probed for specific proteins with enhanced chemiluminescence western blotting detection (GE Healthcare, Marlborough, MA) to probe. For GAS6 stimulation experiments, cells were serum-starved in 1% FBS-DMEM overnight and then stimulated for 30 min with 800 ng/mL recombinant GAS6. Antibodies against AXL, phosphorylated AXL (pAXL; Y702), AKT, phosphorylated AKT (pAKT; S473), ERK, phosphorylated ERK (pERK; T202/T204), MEK1/2, and phosphorylated MEK1/2 (pMEK1/2; S221), were obtained from Cell Signaling Technology (Danvers, MA). An anti–β-actin–horseradish peroxidase antibody was obtained from Santa Cruz Biotechnology (Dallas, TX).

### 2.6 Xenograft experiments

All animal procedures were approved by the Institutional Animal Care and Use Committee at The University of Texas MD Anderson Cancer Center, and all animals received humane care as per the Animal Welfare Act and the “Guide for the Care and Use of Laboratory Animals” from the National Institutes of Health [25]. To create MPNST xenografts, we subcutaneously injected suspensions of MPNST724-NT, MPNST724-AXLsh1, or MPNST724-AXLsh2 cells (2.0 × 10^6^ cells/0.1 mL phosphate-buffered saline) into the flanks of 6-week-old female hairless severe combined immunodeficiency (SCID hairless Outbred SHO-*Prkdc*^*scid*^*Hr*^*hr*^; Charles River, Laboratories, Wilmington, MA) mice (n = 7 mice per construct; 21 mice total). Tumor size was measured twice per week with the use of calipers, and tumor volume (V) was calculated as [V = (length × width×2)/2]. The mice were monitored for tumor burden, body weight, and general well-being.

For experiments assessing the effect of AXL knockdown and pharmacological MEK inhibition on MPNSTs, mice harboring MPNST724-NT or MPNST724-AXLsh2 tumors (mean volume, 100 mm^3^) were treated with vehicle (80 mM citric buffer) via oral gavage daily or the MEK1/2 inhibitor PD0325901 (Selleck Chemicals, Houston, TX; 25 mg/kg) via oral gavage five days per week (n=4 mice per treatment group; 16 mice total). When the vehicle-treated tumors reached a mean volume of 1500 mm^3^, all mice were euthanized, and their tumors were excised, weighed, and frozen or fixed in formalin. Formalin-fixed tissues were subsequently embedded in paraffin for immunohistochemical (IHC) analysis.

For experiments assessing the effect of pharmacological co-inhibition of AXL and MEK on MPNSTs, mice harboring MPNST724 tumors (mean tumor volume, 100 mm^3^) were treated with vehicle (80 mM citric buffer and 70% polyethylene glycol 400), PD0325901 (25 mg/kg), the Met-related inhibitor BMS777607 (Selleck Chemicals, Houston, TX; 25 mg/kg), or both PD0325901 and BMS777607 via oral gavage five days per week (n=5 mice per treatment group; 20 mice total). When the vehicle-treated tumors reached an average volume of 1500 mm^3^, all mice were euthanized, and their tumors were excised, weighed, and frozen or fixed in formalin. Formalin-fixed tissues were subsequently embedded in paraffin for IHC analysis.

### 2.7 Immunohistochemical (IHC) analysis

We performed IHC analysis of xenograft tumor tissue as described previously [26, 27]. The extent of the AXL knockdown was confirmed by IHC analysis. We performed IHC analysis of pMEK and pERK to assess the downstream activation of the MAPK pathway. Expression of Ki67, cleaved caspase 3, TUNEL, and CD34 was analyzed to assess cell proliferation, apoptosis, and angiogenesis, respectively, in xenograft-derived specimens. Ki67 quantification was determined using ImageJ [22].

### 2.8 Statistical analysis

Student’s *t*-tests were used to evaluate differences between experimental groups, and *P* values < 0.05 were considered statistically significant. Data are presented as the mean ± the standard error of the mean unless otherwise indicated. Each experiment was performed in triplicate unless otherwise indicated. Statistical analyses were conducted using the software Prism 6.0 (Graphpad Software, San Diego, CA) or Excel (Microsoft, Redmond, WA).

## 3. Results

### 3.1 GAS6 treatment increased the migratory and invasive capacity of MPNST cells

**Figure 1:**
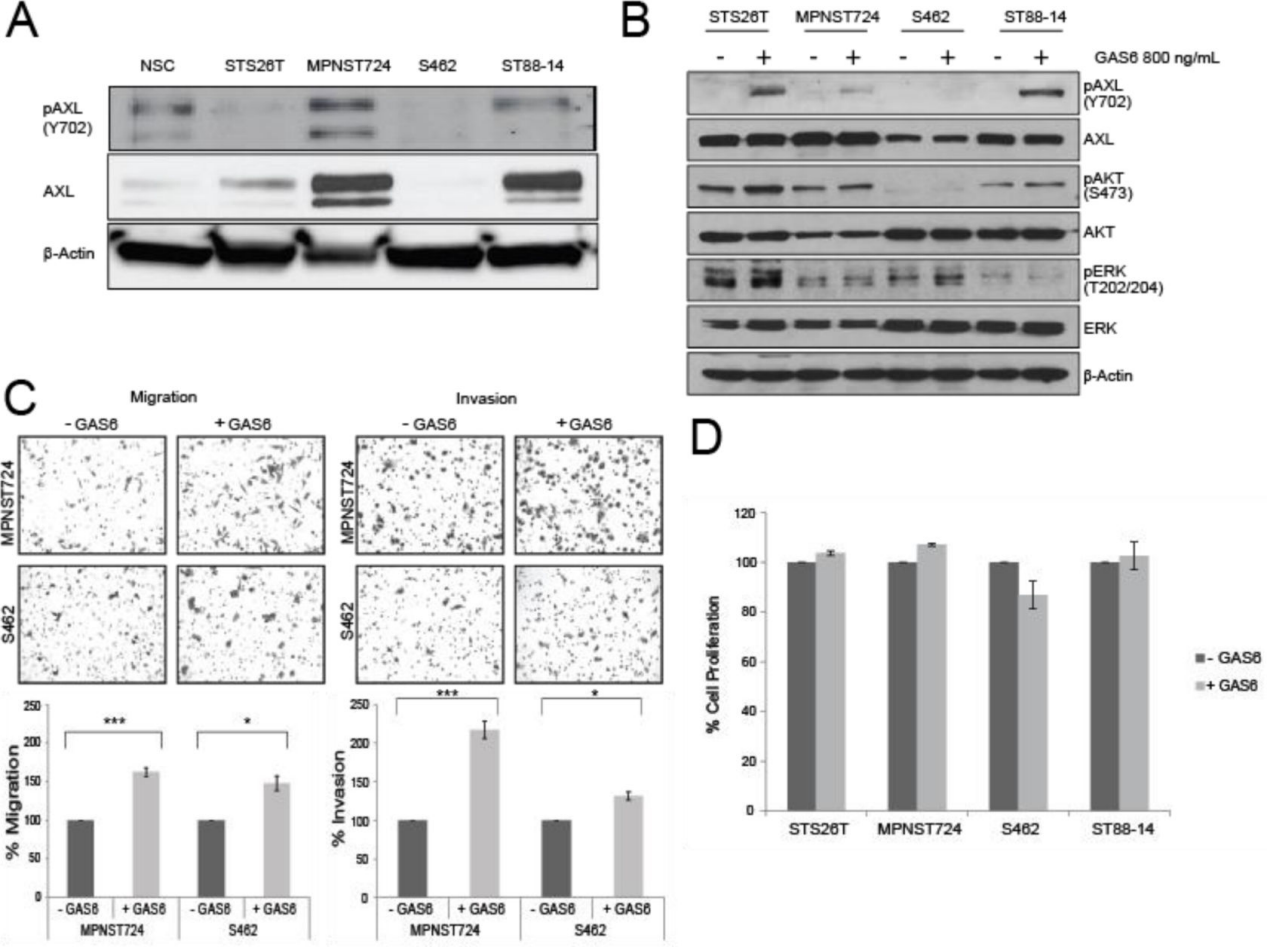
GAS6 stimulates AXL in MPNST. (A) Western blot analysis of MPNST cell lines depicting the expression of AXL and pAXL (Y702). NSC, Normal Schwann cells. (B) Western blot analysis of MPNST cell lines treated with 800 ng/mL GAS6 or left untreated for pAXL, AXL, pAKT, AKT, pERK, and ERK. (C) Upper panel: Representative images from Boyden chamber assays of the migration (left) and invasion (right) of MPNST cells treated with GAS6 or left untreated. Lower panel: Quantification of the migration (right) and invasion (left) cells in the Boyden chamber assays. * *p* < 0.05 and *** *p* < 0.001. (D) MTS assay results showing the percentage of cell proliferation in MPNST cells treated with GAS6 or left untreated.

To determine the role of AXL in MPNST progression, we first assessed the expression and activation status of AXL in MPNST cell lines STS26T, MPNST74, S462, and ST88–14. Variable expression levels of AXL were found among the cell lines. Furthermore, those that had higher expression were also found to have constitutive phosphorylation of AXL at Y702, indicating AXL is activated in some MPNST cells. MPNST724 and ST88–14 cells had higher levels of AXL expression than did normal Schwann cells (Figure 1A), suggesting that AXL and pAXL are differentially expressed in a subset of MPNST cells. The latter is consistent with the heterogenous nature of MPNST tumors.

To determine the effect of AXL stimulation on MPNST cell lines’ tumorigenic potential, cells were assessed following treatment with the AXL ligand GAS6. Compared with untreated MPNST cell lines, those cell lines treated with exogenous GAS6 had increased AXL phosphorylation at Y702 (Figure 1B), by western blotting. The Y702 phosphorylation site was used to show activation dependent on the binding of GAS6 to AXL [15].

The addition of exogenous GAS6 to MPNST cells cultured in low-serum media resulted in a moderate, yet significant increase in cell migration, and invasion (Figure 1C). This finding indicates that when AXL is activated, MPNST cells have greater migratory and invasive properties. In contrast to other sarcoma cells [20], GAS6 treatment did not affect the proliferation of the MPNST cell lines (Figure 1D), measured by MTS assay.

### 3.2 AXL knockdown activated MEK and ERK signaling

**Figure 2:**
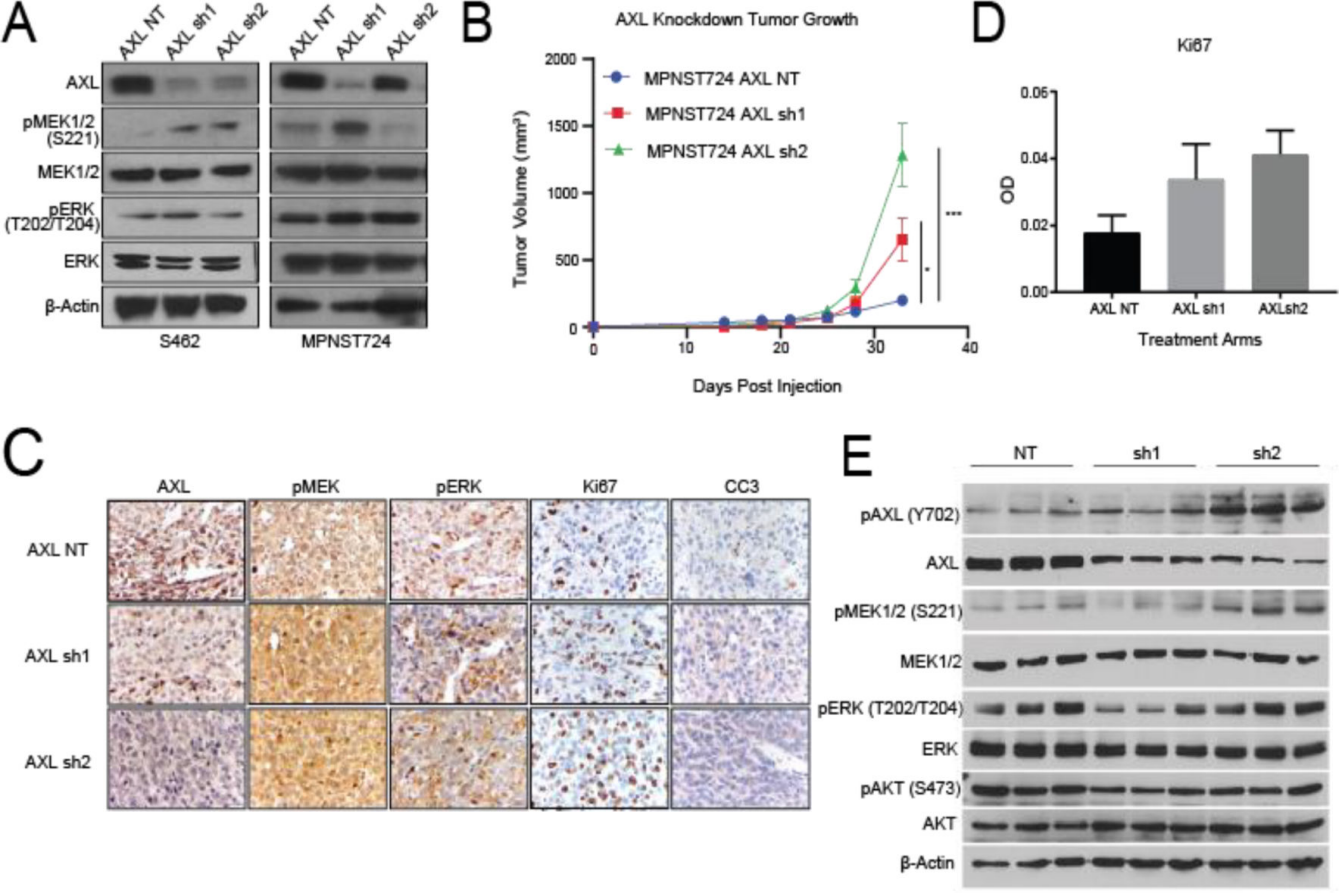
AXL knockdown in MPNST activates the MAPK pathway *in vitro* and *in vivo*. (A) Western blotting of AXL expression after AXL knockdown in S462 and MPNST724 cell lines. (B) Tumor volumes for MPNST724 xenografts (*p* < 0.05 for MPNST724-AXLsh1 and *p* < 0.001 for MPNST724-AXLsh2). (C) Representative images of IHC staining for pMEK1/2, pERK, Ki67, and CC3 expression in MPNST724 xenograft tumor samples (magnification 400x). ***p < 0.001 and * *p* < 0.05. (D) Quantification of Ki67 staining. Bar graph depicts the average of 10 representative fields in three representative animals. (E) Western blot analysis of three representative tumors evaluating the expression of pAXL, AXL, pMEK1/2, MEK1/2, pERK, ERK, pAKT, AKT, and β-Actin.

After observing that stimulation of AXL by GAS6 increased the invasive and migratory properties of MPNST cells, we next examined the effect of AXL knockdown on downstream signaling. Western blot analysis revealed that, in S462 and MPNST724 cells, shRNA-mediated AXL knockdown was accompanied by increased pMEK1/2 expression levels (Figure 2A), suggesting that AXL knockdown may result in compensatory activation of the MAPK pathway. Modest to no increase in pERK was detected by western blot.

To investigate the effect of AXL knockdown on both MPNST tumor growth *in vivo*, we subcutaneously injected MPNST724 cells harboring shNT (non-targeting control), AXL sh1, or AXL sh2 into hairless SCID mice. When AXL was depleted in MPNST724-AXLsh1 and MPNST724-AXLsh2 PDX tumors, they grew more rapidly than the control group (MPNST724-NT tumors) (Figure 2B). Immunohistochemical analysis confirmed lower expression of AXL in the MPNST724-AXLsh1 and MPNST724-AXLsh2 tumors when compared to the control group (MPNST-NT) (Figure 2C). In support of AXL involvement in tumor growth, IHC analysis indicated increased Ki67 staining demonstrating increased cell proliferation in the tumors depleted of AXL (Figure 2D). Further examination of the AXL signaling cascade members demonstrated an increase in pMEK1/2(S221) in MPNST724-AXLsh2 tumors by western blotting (Figure 2E). Such increase was not as evident for the MPNST724-sh1 tumors.

### 3.3 Long-term AXL knockdown enhances MPNST cell sensitivity to MEK1/2 inhibition

**Figure 3:**
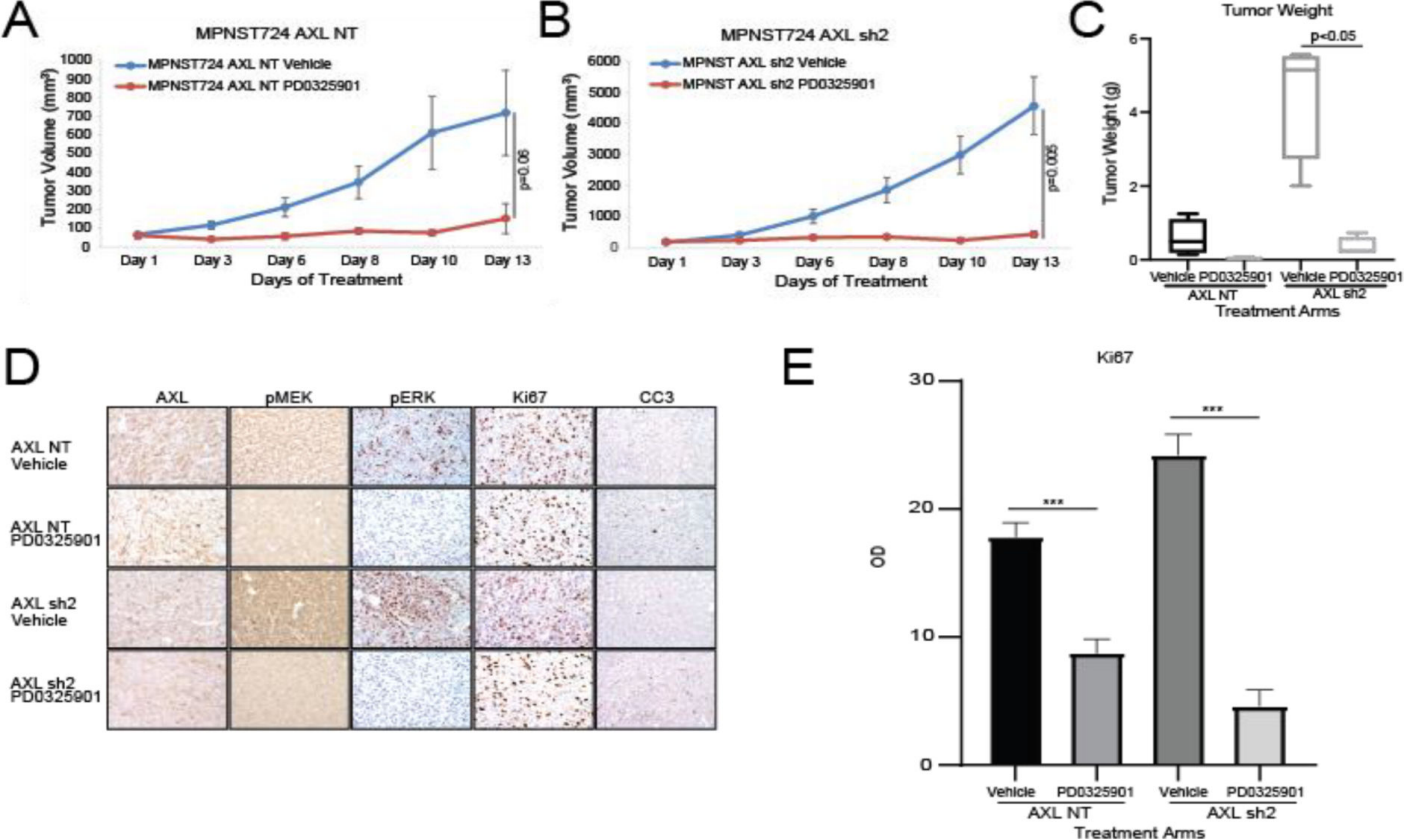
AXL knockdown enhances MPNST sensitivity to MEK1/2 inhibition *in vivo.* AXL knockdown enhances the effects of the MEK inhibitor PD0325901 on the growth of MPNST724- NT xenografts (A) and MPNST724-AXLsh2 xenografts (B). (C) Tumor weights for AXL knockdown xenografts treated with vehicle or PD0325901. (D) IHC analysis of AXL, pMEK1/2, pERK, Ki67, and CC3 expression in MPNST724 xenografts treated with vehicle or PD0325901. (E) Quantification of Ki67 IHC staining. ***p < 0.001.

Based on our *in vitro* experiments and the increased growth of tumors that had long term AXL knockdown, we investigated the expression of both pMEK1/2 and pERK. We observed increased expression of pMEK and pERK in the tumor samples (Figure 2C, 2E). Although we observed a moderate increase in pMEK and pERK1/2 in IHC, a mild increased was confirmed by western blot. Based on these findings, we hypothesized that dual targeting of AXL and MEK1/2 has a stronger antitumorigenic effect than targeting AXL alone. To test this hypothesis, we assessed the growth of MPNST724-NT and MPNST724-AXLsh2 tumors treated with the vehicle or the MEK inhibitor PD0325901. We treated tumors with PD0325901 25 mg/kg/day, as previous studies with this drug ranged from 2.5mg/kg/day to 25mg/kg/day [28, 29]. MPNST724 with AXL knockdown tumors were significantly smaller when treated with MEK inhibitor PD0325901 when compared to the control tumors (Figure 3A and 3B). Tumors for which AXL was knocked down and treated with the MEK inhibitor demonstrated decreased cell proliferation and increased apoptosis as indicated by Ki67 staining and cleaved caspase three (CC3) staining, respectively (Figure 3D and 3E, Supp Figure 1C and 1D). Together, these results suggest that combination of drugs that target both AXL and MEK may be an effective therapeutic strategy for MPNST.

**Figure 4:**
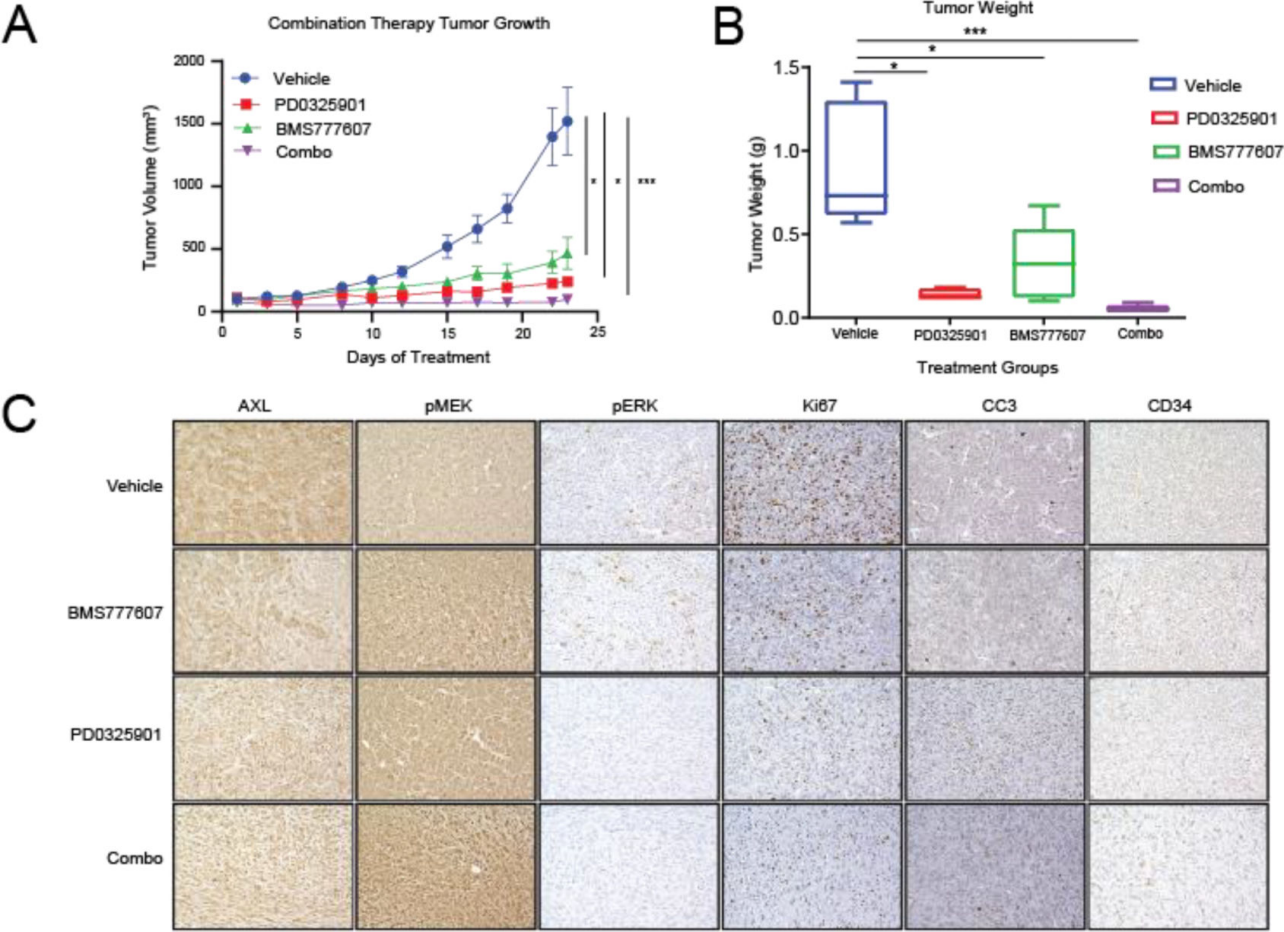
Pharmacological AXL inhibition and MEK inhibition synergistically inhibit MPNST growth *in vivo.* (A and B) MPNST724 xenograft tumors treated with both BMS777607 and PD0325901 were smaller than those treated with either agent alone or with the vehicle. (C) IHC analysis of AXL, pMEK1/2, pERK, Ki67, cleaved caspase 3 (CC3), and CD34 expression in MPNST724 xenografts treated with vehicle, BMS777607 alone, PD0325901 alone, or BMS777607 plus PD0325901 (combo). Magnification 200x. ***p < 0.001 and * *p* < 0.05.

To determine whether these results could be repeated with pharmacological targeting of AXL and MEK1/2, we assessed the growth of MPNST724 tumors in hairless SCID mice treated with vehicle, BMS777607, PD0325901, or both BMS777607 and PD0325901. The inhibitor BMS777607 is a Met family kinase inhibitor, effectively impairing several receptor tyrosine kinases [30]. As expected, tumors treated with the combination therapy were smaller than those treated with either inhibitor alone or with vehicle (Figure 4A and 4B). Furthermore, treatment with both BMS777607 and PD0325901 led to decreased cell proliferation and increased apoptosis, as indicated by Ki67 and cleaved caspase three stainings (Figure 4C and 4D). In agreement with published studies, the levels of pMEK increased upon treatment with PD0325901 (Figure 4D). This phenomenon has also been observed following PD0325901 treatment, with a concomitant decrease in total MEK, in HCT116 colorectal cancer cells as well as in BRAF V600E mutant melanoma cells [31, 32].

## 4. Discussion

Loss of *NF1* is the primary tumor-initiating event in NF1-MPNSTs. NF1 loss has also been noted in sporadic MPNSTs [1, 33]. However, how additional genetic and epigenetic steps alter critical signaling molecules and subsequently promote MPNST progression and metastasis are unknown. Several pathways have been identified as deregulated in MPNST [34, 35]. In this study, we demonstrated that AXL is expressed in some MPNST cell lines. Further, activation of AXL receptor with GAS6 increased promoted invasion and migration. These results suggest that activation of AXL promotes tumor invasion and metastasis in MPNST, which may explain the poor prognosis of these tumors.

Our results showed that the mode of AXL inhibition resulted in different outcomes in our *in vivo* MPNST model. We found that AXL knockdown xenografts eventually grew more than control tumors. Conversely, MPNST PDX tumors treated with the small molecule AXL inhibitor, BMS777607, resulted in decreased tumor volume when compared to the control group. A possible explanation for these conflicting results is that these two methods of AXL inhibition used different mechanisms of action. When AXL was knocked down for a long term by shRNA, a compensatory mechanism was activated. In this case, we observed upregulation of MEK1/2. Torka et al. [36] discovered that small interfering RNA-induced or pharmacological inhibition of AXL increased human epidermal growth factor receptor 3 (HER3) expression and phosphorylation as a compensatory mechanism for the overexpression of AXL, thereby increasing cell viability in MDA-MB-231 (breast) and Ovcar8 (ovarian) cancer cells. Further analysis showed that increased HER3 expression led to AKT phosphorylation [36]. Another study found that the dimerization of HER3 with HER2 activated the PI3K/mTOR and MAPK signaling cascades [37]. Therefore, HER3 upregulation may mediate the compensatory mechanism that is activated in MPNST upon AXL knockdown. Another possible explanation for this discrepancy is that BMS777607 is a Met family kinase inhibitor possessing activity against additional Met family members including Tyro-3, and Mer, Lck, VEGFR-2, and TrkA/B [30]. BMS777607 treatment could have elicited a stronger anti-tumor response through non-specific inhibition of one of these other Met family members. Moreover, BMS777607 acts by competing with ATP for the ATP-binding site of the c-Met kinase domain, keeping the protein in an inactive state [30]. The half-life of BMS777607 when administered intravenously was 4.5 h, 4.8 h, and 4.9 h in mice, rats, and dogs, respectively [30]. The Cmax in humans in a 24 hour period ranged between 18-μg/mL in a fasted state when given 300 mg BID in humans with solid tumors [38]. These data support the notion that effectiveness of the small molecule inhibitor is potentially limited by the pharmacokinetics of the drug, which differs greatly from shRNA-mediated knockdown. Knockdown can last indefinitely for stable cells, such as those generated in the present study. In this case, it is entirely possible that the observed incongruence in effects between the shRNA-mediated knockdown of AXL and the small molecule inhibitor BMS777607 treatment could be driven by 1) the non-specific effect of the small molecule inhibitor on other MET family members 2) the shorter-term dosing of the small molecule inhibitor, compared to the sustained downregulation achieved with shRNAs.

Our studies indicate that combination of an AXL inhibitor and another targeted therapy, such as MEK inhibition, may provide a more effective option than an AXL inhibitor alone in treating MPNSTs. Combination therapy with AXL inhibitors has been found effective for treating various cancers [39, 40]. In the present study, treating MPNST with AXL knockdown in combination with a MEK1/2 inhibitor reduced cell viability, cell proliferation, and tumor growth. These findings agree with the results of several studies in other malignancies reporting that dual targeting of RTKs and MEK is an effective anti-tumor strategy [41, 42]. Hence, a therapy targeting both AXL and MEK1/2 could be an effective treatment for MPNSTs.

MEK1/2 is an attractive co-target for combination therapies in NF1-MPNST. In support of the dual targeting of AXL and MEK1/2, NF1–associated MPNSTs with ERK activation grow more rapidly than do those without ERK activation, suggesting that MEK signaling is an early event in the transformation of neurofibroma into MPNST [43]. Here, we found that MPNST cells with AXL knockdown had increased MEK1/2 and ERK signaling compared with those without AXL knockdown (Figure 3), which agrees with earlier studies’ findings that the overexpression or inappropriate activation of AXL contributes to its transformative capacity [44–46]. In breast and lung cancers, AXL overexpression activates the MAPK and PI3K signaling pathways to increase growth, survival, vascularization, and proliferation [47–50]. Our previous study found that AXL is overexpressed in both human MPNST and neurofibroma samples [14]. The latter suggests that AXL overexpression is an early step in the development of MPNSTs. When we examined the effect of genetic inhibition of AXL combined with pharmacological inhibition of MEK1/2 in MPNSTs, we found that the combination blocked pMEK1/2 and pERK, which in turn slowed the growth of MPNST xenografts. Therefore, inhibiting both AXL and MEK1/2 could be an effective treatment for MPNST patients.

Inhibitors of AXL and MEK are promising anti-cancer therapeutics. Several ongoing pre-clinical studies are investigating the use of AXL inhibitors both as single-agents and as part of dual-agent therapies [51–53]. The AXL-specific inhibitor TP-0903 has been shown to induce apoptosis in B-cell chronic lymphocytic leukemia with effects observed in the nanomolar range [51]. Cabozantinib, an inhibitor that targets several RTKs, is currently in phase II trials for the treatment of urothelial cancer and the treatment of plexiform neurofibromas (NCT04066595; NCT02101736). R428, an AXL-specific inhibitor, is not in clinical trials but has been found in preclinical studies to inhibit the growth and migration of erlotinib-resistant head and neck cancer cells [53] (we did not use R428 in the present study owing to an institutional regulatory obligation to the pharmaceutical company). The MEK1 inhibitor AZD6244 (selumetinib) is currently in phase I/II clinical trial in patients with plexiform neurofibromas (NCT01362803), and phase I and II trials in patients with other cancers (clincaltrials.gov). Preliminary results of the phase I neurofibroma trial showed that 71% of patients with inoperable plexiform neurofibromas had partial responses, and no cases of disease progression were observed [54].

Our findings indicate that dual targeting of AXL and the MAPK pathway inhibits MPNST growth and is a potential therapy for MPNST patients.

## 5. Conclusions

Inhibition of AXL may enhance the sensitivity of MPNST to other targeted therapies; therefore, a therapeutic strategy that includes targeted inhibition of AXL in addition to other targeted or chemotherapeutic agents may result in more potent anti-MPNST effects, and improve the treatment options for MPNST patients.

## Supplementary Material

Supplymentary

## Abbreviations:

MPNST: Malignant peripheral nerve sheath tumors
NF1: neurofibromatosis type 1
RTK: receptor tyrosine kinases
PI3K: phosphatidylinositol 3-kinase
mTOR: mammalian target of rapamycin
TMA: tissue microarray
MEK: mitogen-activated protein kinase kinase
shRNA: short hairpin RNA
ERK: extracellular signal-regulated kinase
FBS: fetal bovine serum
MTS-3: (4,5-dimethylthiazol-2-yl)-5-(3-carboxymethoxyphenyl)-2-(4-sulfophenyl)-2H-tetrazolium
RIPA: radioimmunoprecipitation assay
HER3: human epidermal growth factor receptor 3
PDX: patient-derived xenograft
IHC: immunohistochemical
