# Supplementary material for: AXL Inhibition Enhances MEK Inhibitor Sensitivity in Malignant Peripheral Nerve Sheath Tumors
Source: J Cancer Sci Clin Ther. Author manuscript; Available in PMC 2020 Dec 4. (PMC7717506; doi:10.26502/jcsct.5079091)
Supplement: Supplymentary [file NIHMS1639646-supplement-Supplymentary.pdf]

J Cancer Sci Clin Ther 2020; 4 (3): 189-203 *DOI: 10.26502/jcsct.5079063*

Research Article

AXL Inhibition Enhances MEK Inhibitor Sensitivity in Malignant Peripheral Nerve Sheath Tumors

Sharon M. Landers[1], Angela D. Bhalla[1], XiaoYan Ma[1], Kristelle Lusby[2], Davis Ingram[3], Ghadah Al Sannaa[4], Wei-Lien Wang[3], Alexander J. Lazar[3], Keila E. Torres[1*]

[1]Department of Surgical Oncology, The University of Texas MD Anderson Cancer Center, Houston, TX, USA

[2]Department of Surgery, Division of Plastic Surgery, Indianapolis University School of Medicine, Indianapolis, IN, USA

[3]Department of Pathology, The University of Texas MD Anderson Cancer Center, Houston, TX, USA

[4]Department of Pathology and Genomic Medicine, Houston Methodist Hospital, Houston TX, USA

[*]Corresponding Author: Dr. Keila E. Torres, Department of Surgical Oncology, The University of Texas MD Anderson Cancer Center, Houston, TX, USA, Tel: (713) 792-4242; E-mail: ketorres@mdanderson.org

Received: 12 May 2020; **Accepted:** 04 June 2020; **Published**: 15 June 2020

Citation: Sharon M. Landers, Angela D. Bhalla, XiaoYan Ma, Kristelle Lusby, Davis Ingram, Ghadah Al Sannaa, Wei-Lien Wang, Alexander J. Lazar, Keila E. Torres. AXL Inhibition Enhances MEK Inhibitor Sensitivity in Malignant Peripheral Nerve Sheath Tumors. Journal of Cancer Science and Clinical Therapeutics 4 (2020): 189-203.

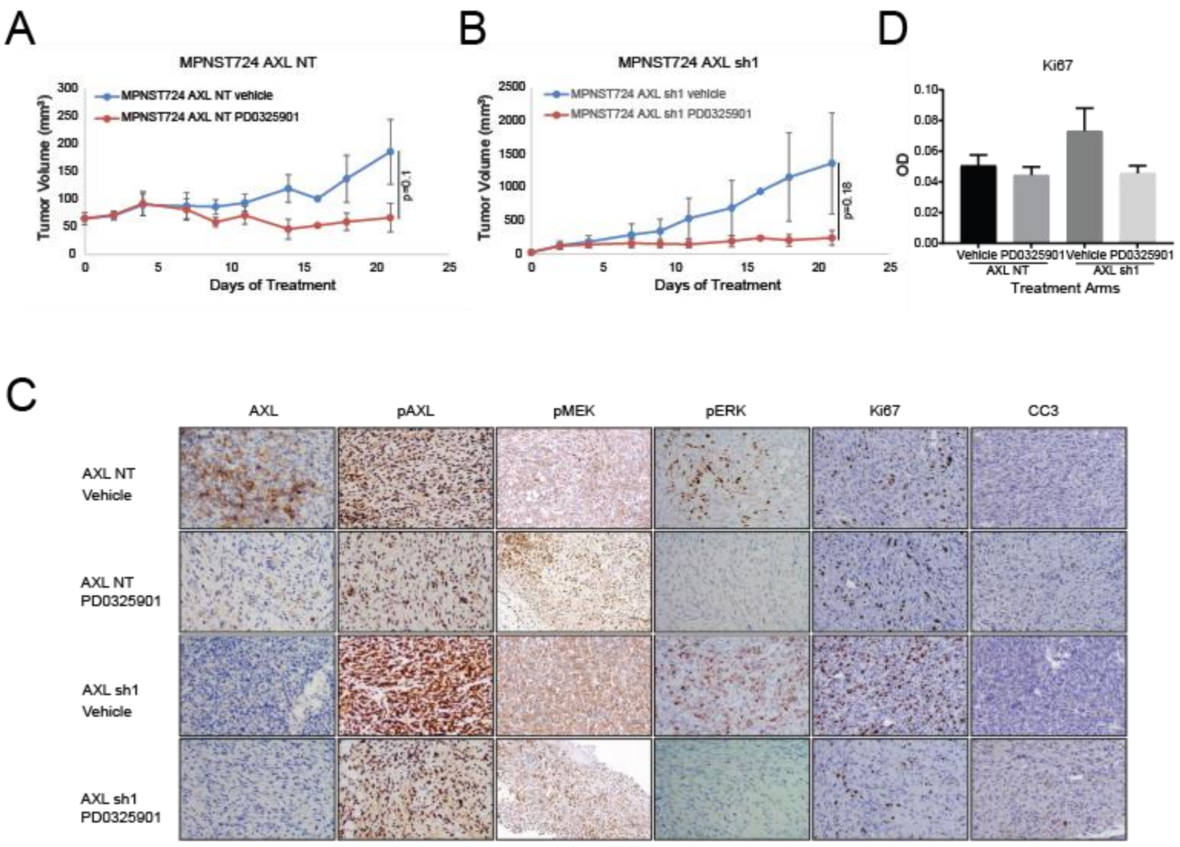

Supplemental Figure 1: AXL knockdown enhances MPNST sensitivity to MEK1/2 inhibition *in vivo*. AXL knockdown enhances the effects of the MEK inhibitor PD0325901 on the growth of MPNST724- NT xenografts (A) and MPNST724-AXLsh1 xenografts (B). (C) IHC analysis of AXL, pAXL, pMEK1/2, pERK, Ki67, and cleaved caspase 3 (CC3) expression in MPNST724 xenografts treated with vehicle or PD0325901. (D) Quantification of Ki-67 IHC staining.

This article is an open access article distributed under the terms and conditions of the Creative Co mMons Attribution (CC-BY) license 4.0
